# Supplementary material for: Tracing and analysis of 288 early SARS-CoV-2 infections outside China: A modeling study
Source: PLoS Med. 2020 Jul 17;17(7):e1003193. doi: 10.1371/journal.pmed.1003193 (PMC7367442; doi:10.1371/journal.pmed.1003193)
Supplement: S1 Text — Modeling traveling cases, delay from arrival to detection and index case detection probability. (PDF) [file pmed.1003193.s001.pdf]

S1 Text. Statistical methods

Modelling traveling cases and delay from arrival to detection

Dataset: The individual data consists of tuples (S, f, o), where:

- S indicates place of departure as Hubei province (H), China other than Hubei (C), outside China (O);
- $f \in \{1, \ldots, T\}$ is the day the case arrived at destination, counted from January 5th up to current date T;
- $o \in \{1, \ldots, T\}$ is the day the case was confirmed, counted from January 5th.

Modelling the detection delay: The difference $D = o - f$ corresponds to the time from arrival to confirmation. Preliminary inspection of the mean and variance of the observed values of D suggested that the coefficient of variation was stable over time and the distribution asymmetrical. We therefore chose a Gamma distribution, which is positive with 2 parameters, and is easily parameterized to yield a constant coefficient of variation - constant shape parameter k corresponding to a coefficient of variation, standard deviation/mean, $1/\sqrt{k}$. We then accounted for changes in detection efficacy, by letting the rate parameter change with time. More precisely, the rate parameter of the distribution was $\beta_f = a * e^{bf}$. We truncated the distribution at $T_D = 25$ days and computed probabilities that D was τ days as:

$$g_f(\tau) = K * \left(P\left(D \leq \tau + 0.5 \middle| \beta_f, k\right) - P\left(D \leq \tau - 0.5 \middle| \beta_f, k\right)\right),$$

where K is a normalization constant accounting for the truncation at T_D.

We denote the corresponding cumulative distribution function of D by $G_f(\tau) = P(D \leq \tau + 0.5 | \beta_f, k)$.

Modelling cases arrival: We computed $A_S = \{A_{S,t}\}_{t=1,\ldots,T}$ the daily number of cases arriving from location S on date t that had been detected before time T, and $N_S = \sum_t A_{S,t}$ the total number of such cases arriving from location S.

Due to the time lag between arrival and confirmation, some cases arriving on time t from location S can be undetected as of time T. We denote $U_{S,t}$ the number of such cases. Then, the total count of cases arriving on day t is given by $A_{S,t} + U_{S,t}$. We assumed a Poisson distribution for this count, $A_{S,t} + U_{S,t} \sim Poisson(I_{S,t})$, where $I_{S,t}$ represents the expected number of imported cases from location S on day t.

We modelled $I_{S,t}$ as a piecewise exponential function in each location of origin S, the exponential growth parameter changing in Hubei after the ban instated on January 23rd and in the rest of China after flight cancellation by major airline companies on January 29th. $I_{S,t}$ was therefore:

$$I_{S,t} = \begin{cases} I_S^{pre} * e^{r_S^{pre} t} & t \leq T_S \\ I_S^{post} * e^{r_S^{post} t} & t > T_S \end{cases} \quad , \quad S = H, C$$

where T_S is the last day *before* the start of quarantine/travel restriction in location S. I_S^{pre} , $r_S^{pre/post}$ are hyperparameters representing the scale and the growth rate of each exponential, and I_S^{post} is determined by continuity of $I_{S,t}$ at T_S.

Outside China we assumed a single exponential function with the same growth rate as in China outside Hubei before travel restrictions were put in place ($r_O = r_C^{pre}$) and a different scale :

$$I_{O,t} = I_O * e^{rot}$$

49 confirmed cases had no information on date of arrival and/or origin of travel. These cases were described with latent variables as follows:

- $A_S^{(M)} = \left\{A_{S,t}^{(M)}\right\}_{t=-T_D+1,\dots,T}$, the time series that accounts for case counts with unknown date of arrival;
- $X_S = \left\{X_{S,t}\right\}_{t=1,\dots,T}$, i.e. case counts with unknown travel origin;
- $X_S^{(M)} = \left\{X_{S,t}^{(M)}\right\}_{t=-T_D+1,\dots,T}$, i.e. cases with both information missing.

The framework described above was extended to account for these cases, i.e. we considered $A_{S,t} + A_{S,t}^{(M)} + U_{S,t} + X_{S,t} + X_{S,t}^{(M)} \sim Poisson(I_{S,t})$ to be the number of cases arriving from destination S on time t.

Likelihood function: The components of the estimated parameters θ and prior distributions are listed in Table A.

Table A. Summary of parameters and their priors.

Parameter	Description	Prior distribution
I_H^{pre}	Scaling factor Hubei	$\log I_H^{pre} \sim Normal(0,1)$
I_C^{pre}	Scaling factor China	$\log I_C^{pre} \sim Normal(0,1)$
I_O	Scaling factor outside China	$\log I_O \sim Normal(0,1)$
r_H^{pre}	Pre-ban growth rate from Hubei	$Normal(0,1)$
r_H^{post}	Post-ban growth rate from Hubei	$Normal(0,1)$
r_C^{pre}	Pre-ban growth rate from China (no Hubei)	$Normal(0,1)$
r_C^{post}	Post-ban growth rate from China (no Hubei)	$Normal(0,1)$
k	Shape parameter in time from arrival to detection distribution	$\chi^2(4)$
a	Scale hyperparameter in time from arrival to detection distribution	$Exponential(1)$
b	Scale hyperparameter in time from arrival to detection distribution	$\log b \sim Normal(0,1)$

The likelihood of the observations is given by:

$$L(\theta) = P(D|\theta, X, A, U)P(A, X, U|\theta)P(\theta)$$

Where:

- $P(A, X, U|\theta)$ is the term describing observed incidence according to the model as:

$$P(A, X, U|\theta) = \prod_{S=H,C,O} \prod_{t=1}^{T} P\left(A_{S,t}, A_{S,t}^{(M)}, X_{S,t}, X_{S,t}^{(M)}, U_{S,t}\middle|\theta\right)$$

$$= \prod_{S=H,C,O} \prod_{t=1}^{T} e^{-I_{S,t}} \frac{I_{S,t}^{A_{S,t}+A_{S,t}^{(M)}+X_{S,t}+X_{S,t}^{(M)}+U_{S,t}}}{\left(A_{S,t} + A_{S,t}^{(M)} + X_{S,t} + X_{S,t}^{(M)} + U_{S,t}\right)!}$$

where $I_{S,t}$ is the expected incidence in location *S* at day *t* described above;

- $P(D|\theta)$ is the term describing observed and unobserved duration between arrival and detection:

$$P(D|\theta,X,A,U) = \prod_{S=H,C,O} \prod_{t=1}^{T} \frac{\left(A_{S,t} + A_{S,t}^{(M)} + X_{S,t} + X_{S,t}^{(M)} + U_{S,t}\right)!}{A_{S,t}!\, A_{S,t}^{(M)}!\, X_{S,t}!\, X_{S,t}^{(M)}!\, U_{S,t}!} [G_t(T - t)]^{U_{S,t}} \prod_{i=1}^{A_{S,t}+A_{S,t}^{(M)}+X_{S,t}+X_{S,t}^{(M)}} g_t(D_{S,t,i})$$

where $D_{S,t,i}$ are the individual times to detection of those travelling from location *S* on day *t,*

- $P(\theta)$ is the prior model for all parameters

$$P(\theta) = P(k)P(a)P(b)P(I_O) \prod_{S=H,C} P(I_S^{pre})P(r_S^{pre})P(r_S^{post})$$

For ease of computation, the likelihood is marginalized over latent variables $A_S^{(M)}$, X_S, $X_S^{(M)}$ and U_S corresponding to cases with missing information $\{A_{S,t}^{(M)}\}$, $\{X_{S,t}\}$, $\{X_{S,t}^{(M)}\}$, $\{U_{S,t}\}$, so that data augmentation is unnecessary in the computation of the posterior distribution for the parameters.

The final likelihood is:

$$\tilde{L}(\theta) = \prod_{S=H,C,O} \left\{ \left[\prod_{t=1}^{T} e^{-I_{S,t} G_t(T-t)} \frac{I_{S,t}^{A_{S,t}}}{A_{S,t}!} \prod_{i=1}^{A_{S,t}} g_t(D_{S,t,i}) \right] \left[\prod_{j=1}^{M_S} \mu_S(o_{S,j}) \right] \right\} \left[\prod_{j=1}^{M_X} \mu(o_{X,j}) \right] \times$$
$$\times \prod_{t=1}^{T} \left[I_t^{X_t} \prod_{k=1}^{X_t} g_t(D_{t,k}) \right] \prod_{S=H,C} P(I_S^{pre})P(r_S^{pre})P(r_S^{post})\, P(I_O)P(k)P(a)P(b).$$

Here we have defined for convenience the following variables: $I_t = \sum_S I_{S,t}$, $\mu_S(t) = \sum_{\delta=t-T_D+1}^{t} I_{S,\delta}\ g(t-\delta|\delta)$ and $\mu(t) = \sum_S \mu_S(t)$ and introduced M_S the number of cases travelling from source S and with unknown date of arrival, X_t is the number of cases that arrived on day t from an unknown source, and M_X is the number of cases with unknown travel source and date of arrival.

Inference was performed by MCMC sampling using *Stan*. We used 3 chains with 6000 iterations and discarded the first 50% as burn-in.

We computed the median of the posterior distributions as well as credible intervals for each parameter in θ. Additionally, we computed predictive distribution statistics about the number of cases confirmed on day t, e.g. the average value as well as upper and lower quantiles, using Poisson distribution with mean $\mu(t) = \sum_{S=H,C,O} \mu_S(t)$.

We compared several model formulations in a sensitivity analysis using the *DIC*. The DIC was computed as $2\,\overline{D(\theta)} - D(\bar{\theta})$, where D is the deviance (D=-2 log Likelihood) and the averages were taken over the posterior distributions.

Modelling index case detection probability

<u>Dataset:</u> We define as seed an imported case or a group of cases that could have started a cluster of local transmission outside China. We computed the number x_1 of transmission clusters where a seed of size 1 was among the cases identified in the cluster and likewise x_2 with seeds of size >1. We also computed the number $\tilde{y}$ of imported cases that did not start a transmission cluster and the number z of clusters for which a seed was not observed among the tested cases, i.e. clusters without a direct link to an imported case.

Modeling index case detection: We assumed that seeds could be of size 1 with probability λ or of size 2 with probability $1 - \lambda$. A seed could be observed with probability π and started a cluster with probability φ.

The number $\tilde{y}$ of imported cases that did not start a cluster consist of y_1 and y_2 seeds of size 1 and 2 such that $y_1 + 2y_2 = \tilde{y}$ and $y_1 + y_2 = y$; however the grouping of these cases is unknown. We computed y out of $\tilde{y}$ using a plug-in estimate where the mean of the fraction y_1/y_2 was $\lambda/(1-\lambda)$, i.e. $y = \tilde{y}/(2-\lambda)$.

Denote w the number of seeds of any size that went undetected and did not give start to a cluster, with probability: $(1-\pi)(1-\varphi)$. w is latent and estimated together with λ, π and φ.

Likelihood function: The likelihood is based on a multinomial distribution for x_1, x_2, y, z and w:

$$L(\theta = (\lambda, \pi, \varphi), w)|x_1, x_2, y, z)$$
$$= \frac{(x_1 + x_2 + y + z + w)!}{x_1!\,x_2!\,y!\,z!\,w!}(\lambda\pi\varphi)^{x_1}((1-\lambda)\pi\varphi)^{x_2}(\pi(1-\varphi))^{y}((1-\pi)\varphi)^{z}((1-\pi)(1-\varphi))^{w}$$

Parameters can be estimated at maximum likelihood:

- Differentiating the likelihood function according to λ, π and φ:

$$\begin{cases} \hat{\lambda} = \dfrac{x_1}{x_1 + x_2} \\ \hat{\pi} = \dfrac{x_1 + x_2 + y}{x_1 + x_2 + y + z + \hat{w}} \\ \hat{\varphi} = \dfrac{x_1 + x_2 + z}{x_1 + x_2 + y + z + \hat{w}} \end{cases}$$

- Approximating the maximum w by looking for the value where $L(\theta, w) = L(\theta, w-1)$ (Pollock KH, Building Models of Capture-Recapture Experiments, The Statistician (1976); 25 (4) : 253-9). We then find:

$$\hat{w} = \frac{(x_1 + x_2 + y + z)(1-\hat{\pi})(1-\hat{\varphi})}{1-(1-\hat{\pi})(1-\hat{\varphi})}$$

By replacing $\hat{\pi}$ and $\hat{\varphi}$ in the previous equation we find that the Maximum Likelihood estimator for w is given by:

$$\hat{w} = \frac{y\,z}{x_1 + x_2}$$

Confidence intervals are computed using profile likelihood methods.

Finally, we estimate the number of unobserved cases that did not give start to a cluster as $\hat{w}(2 - \hat{\lambda})$. The confidence interval on this last quantity is computed by multiplying the confidence intervals of both factors.
