# Supplementary material for: Tracing and analysis of 288 early SARS-CoV-2 infections outside China: A modeling study
Source: PLoS Med. 2020 Jul 17;17(7):e1003193. doi: 10.1371/journal.pmed.1003193 (PMC7367442; doi:10.1371/journal.pmed.1003193)
Supplement: S2 Text — Dataset of international cases, results of likelihood estimation, sensitivity analyses, and analysis of imported clusters. (PDF) [file pmed.1003193.s002.pdf]

S2 Text. Additional results.

Dataset of international cases

We analyze in Fig A the proportion of traveling cases for which we have complete information regarding the timeline of events. Detailed information of the clusters of transmission is reported in Table A.

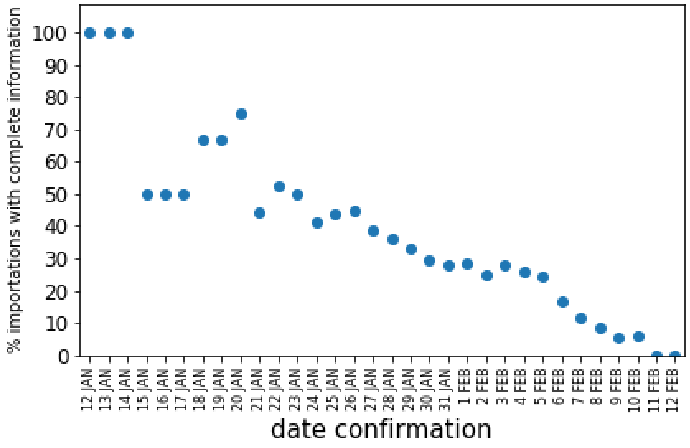

Fig A. Fraction of imported cases with complete information on the timeline of importation.

Table A. Clusters of local transmission

Cluster	Country	ISO3	Size of cluster	Identification of the traveling index case	Index case	Secondary cases	Comments
**cAE01**	United Arab Emirates	ARE		unknown			Available information is not sufficient to reconstruct the details of this cluster
**cCA01**	Canada	CAN	3	not identified or retrospectively identified	CA06 CA07	CA05	CA05 received a visit by Chinese relatives from Wuhan (CA06 CA07) detected only after CA05 tested positive
**cDE01**	Germany	DEU	16	identified prior to cluster detection	DE0	DE01 DE02 DE03 DE04 DE05 DE06 DE07 DE08 DE11 DE12 DE13 DE14 DE15 DE16 ES01	cluster linked to the German company Webasto. DE0 tested positive after the flight back to China, and called the German company to warn about the possible spread of the infection
**cFR01**	France	FRA	2	not identified or retrospectively identified		FR06	medical doctor who treated two Chinese tourists (who tested positive after the flight back to China)
**cFR02**	France	FRA	12	identified prior to cluster detection	GB03	FR07 FR08 FR09 FR10 FR11 GB04 GB05 GB06 GB07 GB08 ES02	GB03 was infected in Singapore (cluster SG02), then traveled to France were he infected 11 British people at a ski resort (5 were detected in France, 5 in the United Kingdom, 1 in Spain)
**cGB01**	United Kingdom	GBR	2	identified prior to cluster detection	GB01	GB02	Chinese mother visited her son, which is a student at the university of York
**cJP01**	Japan	JPN	4	not identified or retrospectively identified		JP06 JP08 JP15	cluster linked to a bus tour in Japan for Chinese tourists. 2 Japanese tour guides and 1 Japanese bus driver have been infected. Two passengers of the bus were later identified as infected travellers from Wuhan
**cJP02**	Japan	JPN	2	not identified or retrospectively identified		JP26	JP26 works at a place visited by Chinese tourists from Hubei
**cJP03**	Japan	JPN	3	unknown		JP30 JP33	JP30 is a taxi driver, JP33 is a member of the family
**cJP04**	Japan	JPN	2	unknown		JP31	JP31 is a surgeon
**cJP05**	Japan	JPN	2	unknown		JP32	
**cJP06**	Japan	JPN	3	unknown		TH20 TH21	TH20 and TH21 do not have travel history to China, bu where locally infected during a trip in Japan
**cJP07**	Japan	JPN	2	unknown		KR12	
**cKR01**	South Korea	KOR	5	identified prior to cluster detection	KR03	KR06 KR10 KR11 KR21	the secondary cases are family members or acquaintances of the index case
**cKR02**	South Korea	KOR	2	identified prior to cluster detection	KR05	KR09	the secondary cases are family members or acquaintances of the index case
**cKR03**	South Korea	KOR	2	identified prior to cluster detection	KR12	KR14	the secondary cases are family members or acquaintances of the index case
**cKR04**	South Korea	KOR	3	identified prior to cluster detection	KR16	KR18 KR22	the secondary cases are family members or acquaintances of the index case
**cKR05**	South Korea	KOR	2	identified prior to cluster detection	KR15	KR20	the secondary cases are family members or acquaintances of the index case
**cKR06**	South Korea	KOR	3	not identified or retrospectively identified	KR26 KR27	KR25	KR26 KR27 traveled from China to visit KR25 and were tested after KR25 was found infected
**cMY01**	Malaysia	MYS	3	identified prior to cluster detection	MY09	MY14 MY17	the secondary cases are family members or acquaintances of the index case
**cSG01**	Singapore	SGP	10	not identified or retrospectively identified		SG19 SG20 SG21 SG24 SG25 SG27 SG28 SG34 SG40	cluster linked to Yong Thai Hang shop, a shop visited by Chinese tourists
**cSG02**	Singapore	SGP	8	not identified or retrospectively identified		SG30 SG36 SG39 KR17 KR19 MY09 GB03	business conference held at Grand Hyatt Singapore (20-22 January)
**cSG03**	Singapore	SGP	2	unknown		SG29	
**cSG04**	Singapore	SGP	5	identified prior to cluster detection	SG08 SG09	SG31 SG33 SG38	cluster linked to The Life Church and Missions of Singapore
**cSG05**	Singapore	SGP	2	unknown		SG32	
**cSG06**	Singapore	SGP	2	unknown		SG35	SG35 is a taxi driver
**cSG07**	Singapore	SGP	2	unknown		SG37	SG37 is private hire car driver
**cSG08**	Singapore	SGP	2	unknown		SG41	
**cSG09**	Singapore	SGP	5	unknown		SG42 SG47 SG52 SG56	cluster linked to Seletar Aerospace Heights construction site
**cSG10**	Singapore	SGP	2	unknown		SG43	
**cSG11**	Singapore	SGP	3	identified prior to cluster detection	SG13 SG26	SG44	SG40 served Quarantine Orders on two suspected individuals (SG13 SG26) who tested positive
**cSG12**	Singapore	SGP	2	unknown		SG46	
**cSG13**	Singapore	SGP	8	unknown		SG48 SG49 SG51 SG53 SG54 SG57 SG58	cluster connected to the Grace Assembly of God church
**cSG14**	Singapore	SGP	3	unknown		SG50 SG55	SG55 is a family member of SG50
**cTH01**	Thailand	THA	2	unknown		TH19	TH19 is a taxi driver
**cTH02**	Thailand	THA	2	unknown		TH22	TH22 is a taxi driver
**cTH03**	Thailand	THA	2	unknown		TH23	TH23 is a taxi driver
**cTH04**	Thailand	THA	3	not identified or retrospectively identified		TH31 TH32	TH31 and TH32 work at a shop visited by Chinese tourists
**cUS01**	United States of America	USA	2	identified prior to cluster detection	US02	US06	US02 traveled to China and infected the partner US06 after getting back to USA
**cUS02**	United States of America	USA	2	identified prior to cluster detection	US10	US11	one member of the couple traveled to China and infected the partner after getting back to USA
**cVN01**	Vietnam	VNM	3	identified prior to cluster detection	VN01	VN02 VN06	VN02 is the son of VN01 while VN06 is the hotel receptionist where VN01 and VN02 stayed
**cVN02**	Vietnam	VNM	7	identified prior to cluster detection	VN05	VN10 VN11 VN12 VN14 VN15 VN16	VN05 was responsible of infecting 3 family members and 1 acquaintance

Results of likelihood estimation

We provide in Table B all parameter estimates and their credible intervals for the baseline scenario (ban on Januay 24) and also for 2 scenarios presented in the sensitivity analyses where the ban was in effect one day earlier or one day after. Results were obtained with 4 chains run for 20000 iterations, discarding the first half as warmup and recording every 10th iteration (thinning) for a total posterior sample size of 4000. For the baseline case, the convergence of the MCMC and the posterior distribution of parameters are shown in Fig B. There was no indication of a lack of convergence. The R.hat (ratio of total variance to within chain variance) was 1 for all parameters, indicating good mixing.

The change in values is commented upon in the sensitivity analysis paragraph below.

Table B. Summary of parameter. Parameter estimates in the baseline scenario (ban on January 24) and two sensitivity analysis scenarios where the ban is effective one day earlier or one day after.

	**Baseline**		**Sensitivity analysis**			
	**Ban 24/1/2020**		**Ban 23/1/2020**		**Ban 25/1/2020**	
**Parameter**	**Median**	**95% Cr. I.**	**Median**	**95% Cr. I.**	**Median**	**95% Cr. I.**
I_H^{pre}	**0.17**	**[0.06, 0.40]**	0.12	[0.04, 0.33]	0.28	[0.12, 0.59]
I_C^{pre}	**0.26**	**[0.11, 0.57]**	0.26	[0.1, 0.59]	0.26	[0.11, 0.57]
I_O	**0.13**	**[0.04, 0.38]**	0.13	[0.04, 0.37]	0.13	[0.04, 0.36]
r_H^{pre}	**0.26**	**[0.21, 0.31]**	0.31	[0.24, 0.38]	0.23	[0.18, 0.29]
r_H^{post}	**-0.64**	**[-0.85, -0.48]**	-0.45	[-0.59, -0.34]	-0.87	[-1.2, -0.61]
r_C^{pre}	**0.04**	**[0.00, 0.08]**	0.06	[0.02, 0.1]	0.06	[0.02, 0.11]
r_C^{post}	**-0.19**	**[-0.54, 0.00]**	-0.21	[-0.53, -0.0]	-0.20	[-0.54, -0.01]
k	**3.35**	**[2.57, 4.34]**	3.38	[2.6, 4.3]	3.34	[2.57, 4.27]
a	**0.21**	**[0.11, 0.33]**	0.21	[0.11, 0.33]	0.20	[0.11, 0.33]
b	**0.03**	**[0.01, 0.06]**	0.03	[0.0, 0.06]	0.03	[0.01, 0.06]

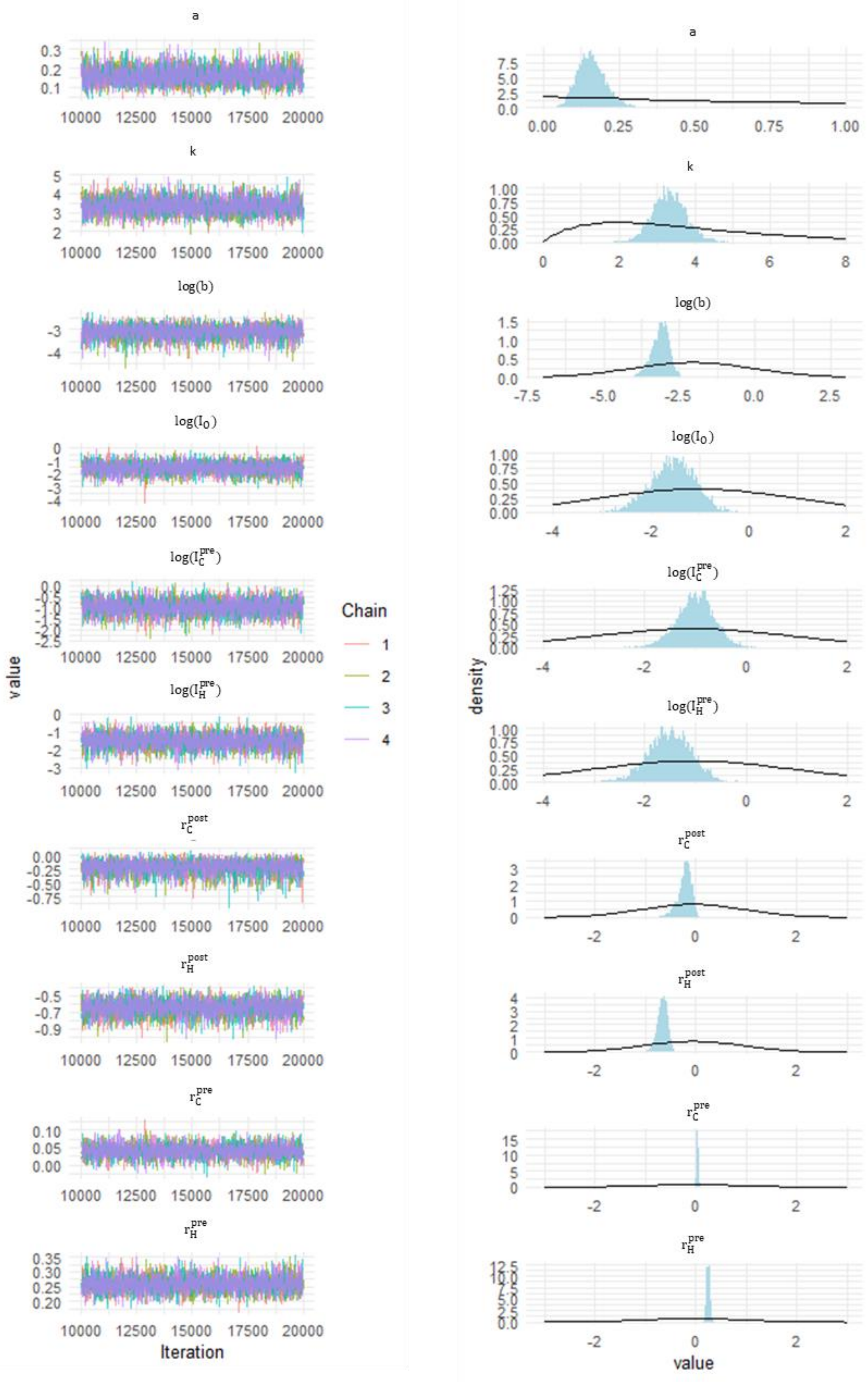

Fig B. Convergence of MCMC procedure. On the left we show the trace of 4 chains for every individual parameter (10000 iterations with 1 in 10 thinning). On the right we plot the corresponding posterior distribution (histogram) and prior distribution (black line).

Sensitivity analyses

On 23/01/2020 all trains, flights and public transports connecting Wuhan with the outside were suspended. We accounted for the possibility that the effect of this ban may have started earlier or may not be completely effective, with measures occurring gradually.

We investigated scenarios in which the effects of the travel ban in Wuhan took place from 2 days before to 2 days after the 24/01/2020. We computed the *DIC* for these models. The model with the ban on the 23/01 had the smallest *DIC* of all, as illustrated in Table C. Introducing the effect of the ban one day earlier led to similar performance.

Table C. DIC of the models with changing assumption on the ban in Wuhan.

Date of ban	DIC
21/01/2020	761.5
22/01/2020	742.7
23/01/2020	741.3
24/01/2020	748.7
25/01/2020	762.9

The corresponding plots for the fit of the number of departing cases and arrivals are shown in Fig C. These show that when the date of effect of the ban is too early, the model fails to capture the number of travelers on January 23rd and 24th, while when it is later the model fails to capture the quick decrease in cases after these dates.

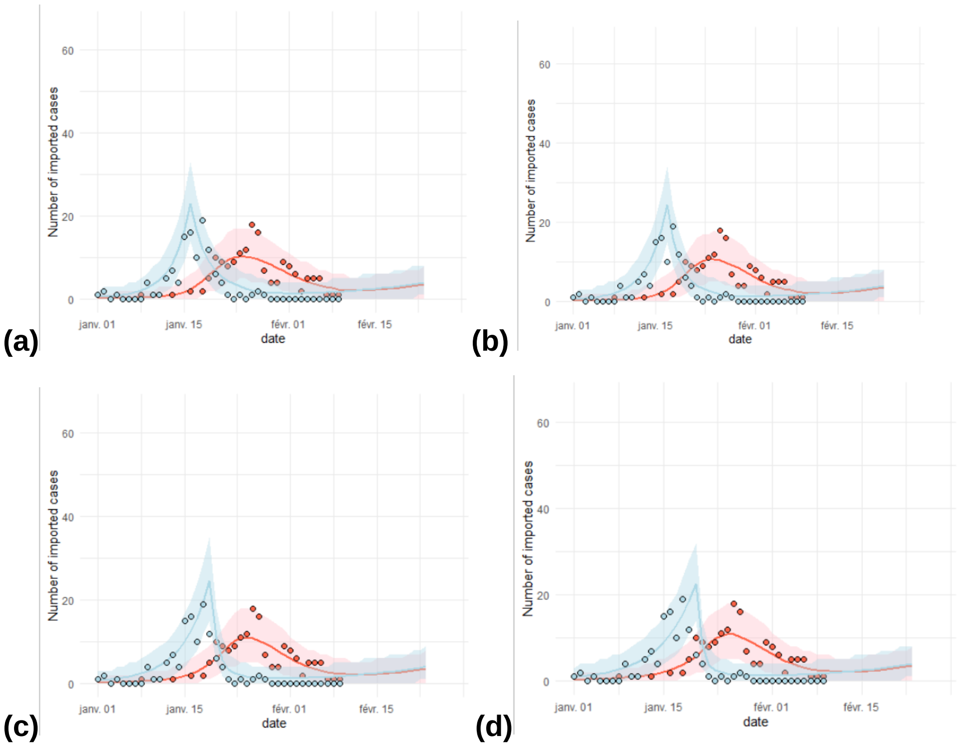

Fig C. Pattern of estimated departures (blue) and reported cases (red) with changing assumption on the ban (a:21/01; b:22/01; c:24/01; d:25/01). The pattern for the 23/01 is shown in the main paper.

Differences in parameter estimates were small (Table B) and in the expected direction: the rate of increase was larger before the date of the ban and smaller afterwards when the effect of the ban was before January 23rd, and it was the other way around when the effect of the ban was delayed.

We also fitted a model where truncation of the delay distribution was after 30 days rather than 25. This did not change the estimates and yielded a similar DIC (742.7 vs. 741.3).

Using a model where the number of travelling cases increased and decreased linearly rather than exponentially before and after the ban date led to a DIC=839.4, larger than the model with an exponential increase. The pattern of arrivals and reported cases shown in Fig D shows that a linear increase did not capture the quick increase in cases travelling in mid-January and supports the choice of an exponential increase.

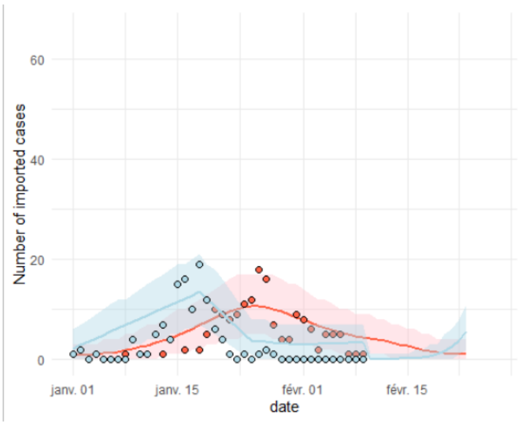

Fig D. Pattern of estimated departures (blue) and reported cases (red) with linear increase and decrease in the number of departures from Hubei, China and other places.

Analysis of imported clusters: summary of parameter estimates

In Table D and Table E we report Maximum Likelihood estimates of parameters in the analysis of imported clusters. We estimate the number of unobserved cases that did not give start to a cluster as $\hat{w}(2-\hat{\lambda})$. The confidence interval on this last quantity is computed by multiplying the confidence intervals of both factors. For $z = 8$ and $z = 27$ we estimate 76 [49, 118] and 255 [186, 369] undetected cases, respectively. We then estimate the fraction of detected imported cases as $(x_1 + 2x_2 + \tilde{y})/[x_1 + 2x_2 + \tilde{y} + (z + \hat{w})(2 - \hat{\lambda})]$, which yields 65% and 36% for $z = 8$ and $z = 27$, respectively.

Table D. Summary of parameter estimates for $x_1 = 13$, $x_2 = 2$, $\tilde{y} = 142$, $z = 8$.

Parameter	Estimate	95% C. I.
λ	0.87	(0.64, 0.98)
π	0.65	(0.59, 0.71)
φ	0.11	(0.07, 0.15)
w	67	(48, 87)

Table E. Summary of parameter estimates for $x_1 = 13$, $x_2 = 2$, $\tilde{y} = 142$, $z = 27$.

Parameter	Estimate	95% C. I.
λ	0.87	(0.64, 0.97)
π	0.36	(0.31, 0.41)
φ	0.11	(0.08, 0.14)
w	225	(182, 272)
