# Supplementary material for: Tracing and analysis of 288 early SARS-CoV-2 infections outside China: A modeling study
Source: PLoS Med. 2020 Jul 17;17(7):e1003193. doi: 10.1371/journal.pmed.1003193 (PMC7367442; doi:10.1371/journal.pmed.1003193)
Supplement: S1 Table — (PDF) [file pmed.1003193.s003.pdf]

S1 Table. Official sources for international cases.

WHO	https://www.who.int/emergencies/diseases/novel-coronavirus-2019/situation-reports/
ECDC	https://www.ecdc.europa.eu/en/geographical-distribution-2019-ncov-cases
Victoria Department of Health	https://www2.health.vic.gov.au/about/media-centre/mediareleases#
Queensland Public Health	https://www.health.qld.gov.au/news-events/health-alerts/novel-coronavirus
Toronto Public Health	https://www.toronto.ca/community-people/health-wellness-care/diseases-medications-vaccines/coronavirus/
Emirates News Agency	https://www.wam.ae/en
Bavarian State Ministry of Health	https://www.stmgp.bayern.de/ministerium/presse/
French Ministry of Health	https://solidarites-sante.gouv.fr/
UK Government Public Health	https://www.gov.uk/health-and-social-care/public-health#news_and_communications
Italian Ministry of Health	http://www.salute.gov.it/portale/nuovocoronavirus/homeNuovoCoronavirus.html
India Ministry of Health	https://mohfw.gov.in/
Japan Ministry of Health	https://www.mhlw.go.jp/index.html
KCDC Press Release	https://www.cdc.go.kr/board/board.es?mid=a30402000000&bid=0030
Malaysia Ministry of Health	http://www.moh.gov.my/index.php/pages/view/349?mid=29
Philippines Department of Health	https://www.doh.gov.ph/
Russian Government	http://government.ru/en/news/
Public Health Agency of Sweden	https://www.folkhalsomyndigheten.se/the-public-health-agency-of-sweden/communicable-disease-control/novel-coronavirus-2019-ncov/
CDC	https://www.cdc.gov/media/dpk/diseases-and-conditions/coronavirus/coronavirus-2020.html
Singapore Ministry of Health	https://www.moh.gov.sg/2019-ncov-wuhan
Thailand Ministry of Public Health	https://pr.moph.go.th/?url=pr/index/2/04
VnExpress Health News	https://vnexpress.net/suc-khoe
