# Supplementary material for: Tracing and analysis of 288 early SARS-CoV-2 infections outside China: A modeling study
Source: PLoS Med. 2020 Jul 17;17(7):e1003193. doi: 10.1371/journal.pmed.1003193 (PMC7367442; doi:10.1371/journal.pmed.1003193)
Supplement: Attachment — Submitted filename: point_by_point_reply.docx [file pmed.1003193.s005.docx]

**Editor**

We would like to thank the editor for the suggestions to improve our manuscript. All point-by-point responses are provided here below.

***Title***

Please revise your title according to PLOS Medicine's style. Your title must be nondeclarative and not a question. It should begin with main concept if possible. "Effect of" should be used only if causality can be inferred, i.e., for an RCT. Please place the study design ("A randomized controlled trial," "A retrospective study," "A modelling study," etc.) in the subtitle (ie, after a colon).

We revised the title as follows: “*Lessons learnt from the first 288 COVID-19 international cases: a modelling study”.*

***Abstract***

Last sentence of the methods and findings section should be a limitation of your methodology.

We added the following closing sentence to the methods and findings section:

*This study is restricted to the early phase of the pandemic, when China was the only large epicenter and foreign countries had not discovered extensive local transmission yet. Missing information in case history represented a limitation of this study and were accounted for through modeling and imputation.*

***Author summary***

At this stage, we ask that you include a short, non-technical Author Summary of your research to make findings accessible to a wide audience that includes both scientists and non-scientists. The Author Summary should immediately follow the Abstract in your revised manuscript. This text is subject to editorial change and should be distinct from the scientific abstract. Please see our author guidelines for more information: <https://journals.plos.org/plosmedicine/s/revising-your-manuscript#loc-author-summary>

We included the Author Summary in the manuscript, after the Abstract section.

***Introduction***

Please update the background information as new evidence has come to light since your submission.

Done.

***Methods***

Since more data are now available, please update the international cases (as possible) in order to validate your model. Please email me at [amisra@plos.org](mailto:amisra@plos.org) to discuss this, as needed.

We additionally collected imported cases in the two-weeks time window of our predictions, relying on WHO situation reports, and extending the original version of this study. This enabled us to add some extra data points to Fig3. Data after February 13 are in line with our predictions, up to February 24. Data in the successive three days diverge from our predictions as they are linked with early detection of multiple imported cases in the Middle East and in Europe, from epidemics occurring in Iran and Italy, respectively. This points to a change in the epidemic situation worldwide, from a single epicenter in China responsible for the majority of exportations worldwide, to a multiple epicenter situation where Iran and Italy mainly contributed to exportations. The situation also needs to be interpreted in light of the controls that were put in place in China since one month already, with heavy travel bans, compared to epidemics that were instead spreading silently and uncontrolled in Iran and Italy up to the first death reports at the end of February. No local or international control measure was put in place in these countries, leading to a large number of cases exported worldwide in a very short amount of time. This prevented the collection of accurate information on such cases.

We were not able to consider additional data on imported cases in the study beyond February 24. This is due to several reasons. First, the change of the epidemic situation, as mentioned before. The epidemic emergency was suddenly recognized in Italy on February 21, 2020, a few days before this study was made available on medRxiv.org. After that time, many countries started to recognize local community transmission that had silently started before. The attention then shifted from importation to local cases, and the overwhelming number of patients in critical conditions requiring hospital and ICU admission. Second, information on imported cases degrades over time. Already at the end of our study period, it was possible to observe an increase in missing information, which we quantified in Figure S1 of the Supporting Information. This is very well known in an ongoing epidemic, as actively collecting information and details on imported cases (dates of travel and symptoms onset, date of COVID-19 confirmation, date of hospital admission, date of case isolation, travel history, epidemiological link with other cases, hospitalization history) requires time and resources and can be done only when these numbers are low and/or when there is a specific interest for understanding epidemiological factors and efficiency of controls at borders - which was the focus of this study. Third, the airline system has been heavily disrupted at the international level, first because of the epicenter in China, and later because of the multiple epicenters in Europe and the Middle East. Many countries put complete travel bans. This inevitably had a strong impact on the pattern of importation, qualitatively changing the epidemic situation that we addressed here. The last 3 points of the additional data we considered in this revision clearly show these effects. Due to the lack of data on airlines and countries adopting targeted or large-scale travel bans, our study, designed to model the travel restrictions to/from China, cannot be extended beyond the end of February, when the pattern of propagation became multicentered and heterogeneous.

The study remains therefore focused on the early stage of the pandemic. Its findings, however, become again critical in the current situation to prevent second waves, now that countries have reduced their epidemic activity and progressively phase out lockdown.

This is now thoroughly discussed in the Discussion section.

Please specify the sources of your data in the methods section providing citations instead of placing this in an SI file.

Done.

Please add the following statement, or similar, to the Methods: "This study is reported as per the Strengthening the Reporting of Observational Studies in Epidemiology (STROBE) guideline (SChecklist)."

Done. The statement has been added at the end of the paragraph “Data collection and synthesis”, in the methods section.

***Discussion***

Please present and organize the Discussion as follows: a short, clear summary of the article's findings; what the study adds to existing research and where and why the results may differ from previous research; strengths and limitations of the study; implications and next steps for research, clinical practice, and/or public policy; one-paragraph conclusion.

We reorganized the Discussion session as requested and to allow the inclusion of the additional points of discussion raised by the reviewers.

Please revise the language in this section esp lines 184-190, line 206-209. You may wish to expand on local transmission, underdetection and undiagnosis of cases worldwide here.

We revised the language and discussed the role of Italy and Iran as novel epicenters of the pandemic.

Please replace epidemic with pandemic.

Done.

Please comment on second waves of infections as imported cases are rising in China.

We included a paragraph in the discussion section. “*Monitoring imported cases is critical in territories where the attention is concentrated on preventing the introduction of cases. At the time of writing, countries such as China and Australia show limited local transmission and are concentrating strong efforts in border controls to avoid a second epidemic wave, that could be fueled by the large proportion of the population still susceptible to SARS-CoV2. Strict measures, such as massive testing and quarantine of incoming travelers, are being implemented to block silent introductions of cases as happened at the beginning of the pandemic.*”

And at the end of the paper: *“Our findings provide critical epidemiological understanding on the rate of importation and detection of cases during the early phase of the pandemic. Though effective in reducing international spread, the travel ban in Wuhan did not prevent the seeding of the pandemic in other countries, later becoming new epicenters of the pandemic. The epidemiological features of COVID-19 facilitated the silent spread of the epidemic in seeded countries. The lessons learnt during the early phase of the pandemic become again critical to prevent second waves, now that countries have reduced their epidemic activity and progressively phase out lockdown.”*

***Supporting information***

Please ensure that the study is reported according to the STROBE guideline, and include the completed STROBE checklist as Supporting Information. Please add the following statement, or similar, to the Methods: "This study is reported as per the Strengthening the Reporting of Observational Studies in Epidemiology (STROBE) guideline (SChecklist)." The STROBE guideline can be found here: <http://www.equator-network.org/reporting-guidelines/strobe/>. When completing the checklist, please use section and paragraph numbers, rather than page numbers.

In most ways, this study is a case series analysis for which EQUATOR does not provide checklists. However, we used the general STROBE statement as a blueprint to show that we complied with good practices regarding description of purpose, population, methods and interpretation. The checklist has been completed and included as Supporting Information.

**Reviewer #1**

We would like to thank the reviewer for describing our manuscript as novel and of interest, as well as for the suggestions provided that helped us improve the presentation of our study. All point-by-point responses are provided here below.

***Language***

**1)** The language in this paper needs to be reframed as analysis of the early stages of an outbreak rather than a description of an ongoing and changing situation. Current sections read somewhat like a news article rather than journal article. Many of these problems can be fixed by changing tense.

Done.

**3)** Several sections were difficult to read or had grammatical errors.

13 "We characterized importations timeline..."

Corrected. And we took care of checking the entire ms to improve its readability.

76 The definition in this section is confusing. Here S = H, C while in the supplementary information (which is clearer) S = H, C, O. Perhaps include S on the second line of the piecewise equation where S = O?

We followed the reviewer’s suggestion and wrote the second equation in terms of S, where S=O.

86 It is hard to read subsequent sentences where one ends and the other starts with a variable or function name.

This has been corrected in the article.

98-101 In "Estimation of under-detection of imported cases" it is not clear what you are saying here or how the multinomial is defined. The next part of the section (where you define the likelihood) clarifies but the intro in lines 98-101 is confusing:
"We modelled the number of such 'cluster seeds', i.e. groups of index cases, with a multinomial distribution depending on the portion of cluster seeds of size 1 or greater than 1 (for simplicity, this was taken as 2), on the probability of detection of a seed, and on occurrence of secondary transmission."

We expanded the section by adding details in order to avoid any confusion. “*We analyzed clusters of transmission generated by imported cases (index case(s) in each cluster) to estimate undetected importations. A cluster can be seeded by more than one index case, e.g. by infected family members who travelled together. The number of clusters of local transmission was modelled with a multinomial distribution according to the number of index cases and whether these were imported cases. As detailed in the Supporting Information, the likelihood function was a function of four observable quantities: the number of observed clusters with one imported index case (*$x_{1}$*); the number of observed clusters with more than 1 imported index case (*$x_{2}$*); the number of known imported cases causing no onward transmission (*$\tilde{y}$*); the number of clusters for which an index case was not identified (*$z$*); and a fifth and unobserved quantity, the number of undetected imported cases who did not start onward transmission (*$w$*). Maximization of the likelihood allowed estimating w from the records of* $x_{1}$*,* $x_{2}$*,* $\tilde{y}$*,* $z$ *and to compute the expected number of unobserved importation cases. Additional details are described in the Supporting Information (S1 Text. Statistical methods).*”

197-198 What has an ability of 36%?
"Our estimates indicate an ability of 36% to detect imported cases in countries outside China."

We changed the sentence as follows: "*Our estimates indicate a detection ability of 36% to ascertain imported cases in countries outside China*."

199 This reads poorly (perhaps "Previous detection rate estimates..." ?)
"Previous estimates range from 27% [13] to 38% [13, 15] detection rates, with variations across
200 countries [13, 15]."

We corrected the sentence following the reviewer’s suggestion.

***References***

**2)** There are not enough references from primary sources - this paper should be framed around existing scientific knowledge from previous outbreaks. About a third of the references are from primary sources (published articles or books), another third are from organisational reports or medRxiv and the last third are from online sources (e.g. Twitter, online news).

We updated 7 medRxiv citations with their corresponding published articles, which were not yet available at the time of submission of the manuscript. References from organisational reports and online sources are cited here as the study deals with an ongoing epidemic. This has 2 effects. On one side, scientific knowledge becomes available in the form of preprints for rapid dissemination, in advance of academic publication that can take some time. The scientific community has therefore heavily been using bioRxiv and medRxiv as reference archives for COVID-19 epidemic. On the other side, given that our study focused on imported cases, it had to rely on online sources to extract the type and detail of information needed for each imported case to conduct the analysis. This was not available from primary sources, as our paper was (and still is) the first to report on the study of imported cases at length. Our preprint and the link to the db has become the reference study for imported cases.

Additionally, a couple citations appear to be incomplete.
----
S. Bhatia et al., "Report 6: Relative sensitivity of international surveillance," p. 6.

Corrected.

***Methods***

**4)** There is a section with a sensitivity analysis where the ban date in Wuhan is a day later. It would be nice to have model comparison using LOO or WAIC between a few select models (piecewise vs single exponential, etc...) Your model should explain the data better than a naive model and it would be easy to show here.

As noted by Vehtari & al regarding computing the LOO, “The situation is … complicated for *non-factorizable* models” as “… computing the pointwise log-likelihood for non-factorizable models is often impossible,” (http://mc-stan.org/loo/articles/loo2-non-factorizable.html). In our model, the likelihood does not factorize as a product over individual cases contributions.

Therefore we resorted to computation of the DIC, which, despite its limitations, may offer some indication to the fit of the model. We now report in the Supporting Information the DIC for models where the date of the ban is changed for 2 days before up to 2 days after January 23; we also report a model where the number of exported cases changes linearly with time before and after the ban. These results show that the model with an exponentially changing number of cases and ban on January 23^rd^ had the smallest DIC.

***Figures***

Fig S2:

a) The MCMC chains appear to be thinned; this should be noted in the caption.

b) You should state if the mixing in STAN reported any divergence if you are showing convergence of the chains.

c) You state that both the black line and histogram show the posterior distribution, but the black lines look a bit more like priors to me.

a) we took the opportunity to increase the number of iterations. We now report that we used 4 chains run for 20000 iterations, with 50% discarded as warm-up and 1 in 10 thinning, for a total posterior sample size of 4000.

b) There was no indication that the chains did not converge (Rhat was 1 for all parameters). No divergent steps were reported.

c) The black line is indeed the prior distribution, this has been corrected.

***Additional data for analysis***

**5)** I realise the current COVID-19 pandemic is ongoing and any additional analysis will not be comprehensive.  But given that there is ongoing data, the paper would benefit from comparing model predictions to a more current set of data.

We report here the response to the Editor on the same point.

We additionally collected imported cases in the two-weeks time window of our predictions, relying on WHO situation reports, and extending the original version of this study. This enabled us to add some extra data points to Fig3. Data after February 13 are in line with our predictions, up to February 24. Data in the successive three days diverge from our predictions as they are linked with early detection of multiple imported cases in the Middle East and in Europe, from epidemics occurring in Iran and Italy, respectively. This points to a change in the epidemic situation worldwide, from a single epicenter in China responsible for the majority of exportations worldwide, to a multiple epicenter situation where Iran and Italy mainly contributed to exportations. The situation also needs to be interpreted in light of the controls that were put in place in China since one month already, with heavy travel bans, compared to epidemics that were instead spreading silently and uncontrolled in Iran and Italy up to the first death reports at the end of February. No local or international control measure was put in place in these countries, leading to a large number of cases exported worldwide in a very short amount of time. This prevented the collection of accurate information on such cases.

We were not able to consider additional data on imported cases in the study beyond February 24. This is due to several reasons. First, the change of the epidemic situation, as mentioned before. The epidemic emergency was suddenly recognized in Italy on February 21, 2020, a few days before this study was made available on medRxiv.org. After that time, many countries started to recognize local community transmission that had silently started before. The attention then shifted from importation to local cases, and the overwhelming number of patients in critical conditions requiring hospital and ICU admission. Second, information on imported cases degrades over time. Already at the end of our study period, it was possible to observe an increase in missing information, which we quantified in Figure S1 of the Supporting Information. This is very well known in an ongoing epidemic, as actively collecting information and details on imported cases (dates of travel and symptoms onset, date of COVID-19 confirmation, date of hospital admission, date of case isolation, travel history, epidemiological link with other cases, hospitalization history) requires time and resources and can be done only when these numbers are low and/or when there is a specific interest for understanding epidemiological factors and efficiency of controls at borders - which was the focus of this study. Third, the airline system has been heavily disrupted at the international level, first because of the epicenter in China, and later because of the multiple epicenters in Europe and the Middle East. Many countries put complete travel bans. This inevitably had a strong impact on the pattern of importation, qualitatively changing the epidemic situation that we addressed here. The last 3 points of the additional data we considered in this revision clearly show these effects. Due to the lack of data on airlines and countries adopting targeted or large-scale travel bans, our study , designed to model the travel restrictions to/from China, cannot be extended beyond the end of February, when the pattern of propagation became multicentered and heterogeneous.

The study remains therefore focused on the early stage of the pandemic. Its findings, however, become again critical in the current situation to prevent second waves, now that countries have reduced their epidemic activity and progressively phase out lockdown.

This is now thoroughly discussed in the Discussion section.

**Reviewer #2**

We would like to thank the reviewer for considering our manuscript to be important, and to provide useful insights on the methods. All point-by-point responses are reported here below.

***Supporting Information***

The mathematics is fully documented in supplemental materials and will seem complex but fundamentally - this is largely modelling growth functions with a structured break due to travel bans over a period of time. Latent variables were used to incorporate missing data where cases had incomplete information, authors used an inference technique to marginalise ("summing out" the probability of a random variable given the joint probability distribution). The Bayesian framework means that there are quite a few distributions to fit around model parameters. A slight nit-pick of this would be that the supplemental could potentially better rationalise the authors assumption on the choice of distribution. It may seem familiar to those with mathematical modelling experience, but it would not likely to be obvious to most. For instance, gamma distribution skewed nature to reflect change in surveillance; also rationale needed to truncate the gamma distribution on the detection delay to 25 days.

We introduced a few lines in the Supporting Information commenting on the choice of the gamma distribution, and provided a sensitivity analysis with truncation at 30 days.

*“Preliminary inspection of the mean and variance of the observed values of D suggested that the coefficient of variation was stable over time and the distribution asymmetrical. We therefore chose a Gamma distribution, which is positive with 2 parameters, and is easily parameterized to yield a constant coefficient of variation.”*

Line 25 in Supplemental - I think the authors meant "tuples" here

Corrected.

***Additional analysis***

The rebound in imported cases in China (and other areas) is now occurring so it would be interesting to conduct some follow-up work given how fast paced new data is coming in.

We report here the response to the Editor and Reviewer #1 on the same point.

We additionally collected imported cases in the two-weeks time window of our predictions, relying on WHO situation reports, and extending the original version of this study. This enabled us to add some extra data points to Fig3. Data after February 13 are in line with our predictions, up to February 24. Data in the successive three days diverge from our predictions as they are linked with early detection of multiple imported cases in the Middle East and in Europe, from epidemics occurring in Iran and Italy, respectively. This points to a change in the epidemic situation worldwide, from a single epicenter in China responsible for the majority of exportations worldwide, to a multiple epicenter situation where Iran and Italy mainly contributed to exportations. The situation also needs to be interpreted in light of the controls that were put in place in China since one month already, with heavy travel bans, compared to epidemics that were instead spreading silently and uncontrolled in Iran and Italy up to the first death reports at the end of February. No local or international control measure was put in place in these countries, leading to a large number of cases exported worldwide in a very short amount of time. This prevented the collection of accurate information on such cases.

We were not able to consider additional data on imported cases in the study beyond February 24. This is due to several reasons. First, the change of the epidemic situation, as mentioned before. The epidemic emergency was suddenly recognized in Italy on February 21, 2020, a few days before this study was made available on medRxiv.org. After that time, many countries started to recognize local community transmission that had silently started before. The attention then shifted from importation to local cases, and the overwhelming number of patients in critical conditions requiring hospital and ICU admission. Second, information on imported cases degrades over time. Already at the end of our study period, it was possible to observe an increase in missing information, which we quantified in Figure S1 of the Supporting Information. This is very well known in an ongoing epidemic, as actively collecting information and details on imported cases (dates of travel and symptoms onset, date of COVID-19 confirmation, date of hospital admission, date of case isolation, travel history, epidemiological link with other cases, hospitalization history) requires time and resources and can be done only when these numbers are low and/or when there is a specific interest for understanding epidemiological factors and efficiency of controls at borders - which was the focus of this study. Third, the airline system has been heavily disrupted at the international level, first because of the epicenter in China, and later because of the multiple epicenters in Europe and the Middle East. Many countries put complete travel bans. This inevitably had a strong impact on the pattern of importation, qualitatively changing the epidemic situation that we addressed here. The last 3 points of the additional data we considered in this revision clearly show these effects. Due to the lack of data on airlines and countries adopting targeted or large-scale travel bans, our study , designed to model the travel restrictions to/from China, cannot be extended beyond the end of February, when the pattern of propagation became multicentered and heterogeneous.

The study remains therefore focused on the early stage of the pandemic. Its findings, however, become again critical in the current situation to prevent second waves, now that countries have reduced their epidemic activity and progressively phase out lockdown.

This is now thoroughly discussed in the Discussion section.

***Discussion***

One final issue is potentially, which the authors do not address in the discussion is the impact of inaccuracies of testing. Some studies have characterised multiple shedding routes (<https://www.ncbi.nlm.nih.gov/pubmed/32065057?dopt=Abstract>). Accurate testing methods are work in progress but it's worth noting that when new studies do come out on these parameters, it would be worth incorporating potential false negative results which may have on the impact of rebounding infection rates from imported cases.

We fully agree that importation cases had to test positive to be identified as such and enter our roster. RT-PCR tests have imperfect sensitivity, for example due to swabbing or to the timing of sampling relative to infection. We report this limitation in the discussion acknowledging that some cases could have tested negative, which is also very consistent with our finding that only 36% of imported cases could have been recorded.

We put in the manuscript: *“Some cases tested for SARS-CoV-2 could have been falsely negative, and this would affect both analyses presented in the manuscript. This is in line with estimating that a large portion of imported cases were not identified.”*
